# Left Ventricular Mechanics Differ in Subtypes of Aortic Stenosis Following Transcatheter Aortic Valve Replacement

**DOI:** 10.3389/fcvm.2021.777206

**Published:** 2022-01-17

**Authors:** Adil Wani, Daniel R. Harland, Tanvir K. Bajwa, Stacie Kroboth, Khawaja Afzal Ammar, Suhail Q. Allaqaband, Sue Duval, Bijoy K. Khandheria, A. Jamil Tajik, Renuka Jain

**Affiliations:** ^1^Aurora Cardiovascular and Thoracic Services, Aurora Sinai/Aurora St. Luke's Medical Centers, University of Wisconsin School of Medicine and Public Health, Milwaukee, WI, United States; ^2^Advocate Aurora Research Institute, Advocate Aurora Health, Milwaukee, WI, United States; ^3^Cardiovascular Division, University of Minnesota Medical School, Minneapolis, MN, United States

**Keywords:** aortic stenosis, echocardiography, global longitudinal strain, transcatheter aortic valve replacement, valve disease

## Abstract

**Background:**

Left ventricular (LV) mechanics are impaired in patients with severe aortic stenosis (AS). We hypothesized that there would be differences in myocardial mechanics, measured by global longitudinal strain (GLS) recovery in patients with four subtypes of severe AS after transcatheter aortic valve replacement (TAVR), stratified based upon flow and gradient.

**Methods:**

We retrospectively evaluated 204 patients with severe AS who underwent TAVR and were followed post-TAVR at our institution for clinical outcomes. Speckle-tracking transthoracic echocardiography was performed pre- and post-TAVR. Patients were classified as: (1) normal-flow and high-gradient, (2) normal-flow and high-gradient with reduced LV ejection fraction (LVEF), (3) classical low-flow and low-gradient, or (4) paradoxical low-flow and low-gradient.

**Results:**

Both GLS (−13.9 ± 4.3 to −14.8 ± 4.3, *P* < 0.0001) and LVEF (55 ± 15 to 57 ± 14%, *P* = 0.0001) improved immediately post-TAVR. Patients with low-flow AS had similar improvements in LVEF (+2.6 ± 9%) and aortic valve mean gradient (−23.95 ± 8.34 mmHg) as patients with normal-flow AS. GLS was significantly improved in patients with normal-flow (−0.93 ± 3.10, *P* = 0.0004) compared to low-flow AS. Across all types of AS, improvement in GLS was associated with a survival benefit, with GLS recovery in alive patients (mean GLS improvement of −1.07 ± 3.10, *P* < 0.0001).

**Conclusions:**

LV mechanics are abnormal in all patients with subtypes of severe AS and improve immediately post-TAVR. Recovery of GLS was associated with a survival benefit. Patients with both types of low-flow AS showed significantly improved, but still impaired, GLS post-TAVR, suggesting underlying myopathy that does not correct post-TAVR.

## Introduction

Aortic valve (AV) replacement in patients with symptomatic severe aortic stenosis (AS) improves survival and has the potential to reverse left ventricular (LV) systolic dysfunction (1, 2). Mortality following surgical valve replacement (3) and transcatheter AV replacement (TAVR) (4) is higher in the subset of patients with low-flow severe AS; in this subset, LV systolic dysfunction can be due to both increased afterload from the stenotic valve (which is reversible) and intrinsic myopathy (which may not be reversible). Despite higher procedural mortality in low-flow severe AS, these patients still demonstrate improved longevity with AV replacement compared with medical management. There is now greater awareness that there are not two, but four subtypes of severe AS, based upon flow and mean gradient, and that these subtypes may have different patterns of myocardial recovery and long-term clinical outcomes (5–7).

Myocardial recovery traditionally has been assessed as improvement in LV ejection fraction (LVEF) after AV replacement. LVEF has limitations as a marker of LV function as it represents the gross change in LV volume and fails to encompass complex, subtle structural changes in the LV. Speckle-tracking has emerged as a useful technology to detect subclinical LV dysfunction. Strain, a dimensionless measure of deformation of a solid object, is a novel technique that allows assessment of segmental myocardial deformation. Longitudinal strain has been shown to be more sensitive than LVEF in the detection of subclinical myocardial dysfunction in severe AS (8, 9). Global longitudinal strain (GLS) has been noted to improve after surgical AVR, and lack of improvement has been associated with adverse clinical outcomes (10, 11). GLS has also been shown to improve post TAVR (12) in patients with severe AS independent of LVEF. Although the relationship of GLS recovery to clinical outcomes has been previously studied (10, 13), the relationship to the four AS subtypes is unknown.

We investigated myocardial recovery in a large series of patients with symptomatic severe AS who have undergone TAVR, classifying them according to four AS subtypes (14). We hypothesized that GLS recovery was different across AS subtypes and that this would correlate with clinical outcomes.

## Materials and Methods

### Patient Population

We studied 204 patients with symptomatic severe AS who underwent TAVR from 2012 to 2016. Patients were referred to our high-volume tertiary care center and evaluated by the Structural Heart Program, composed of interventional echocardiographers, interventional cardiologists, cardiac surgeons, and nurse practitioners.

Patients were selected for TAVR based upon transthoracic echocardiography (TTE) criteria for severe AS in combination with a formal Heart Valve Team assessment. TAVR was performed in patients with symptomatic severe AS who were deemed at high or inoperable risk for surgical valve replacement. Patients were excluded from this study if they underwent valve-in-valve procedures for prior bioprosthetic AV replacement degeneration or had poor transthoracic image quality that precluded assessment of GLS either pre- or post-TAVR. All patients who had TTE available pre-TAVR and post-TAVR (either prior to discharge or up to one month after) with GLS performed were included in this analysis.

Clinical variables including age, sex, and comorbidities were queried from the electronic medical chart (EPIC Systems, Madison, WI), as well as long-term outcomes at one year post-TAVR including congestive heart failure hospitalization and death. Additional echocardiographic variables were collected pre-TAVR and post-TAVR. This study was approved by the Aurora Health Care Institutional Review Board. As this was a retrospective study, the requirement for written, informed consent was waived.

### Echocardiographic Data

TTE was performed pre-TAVR, prior to hospital discharge, and 1 month after the procedure using GE Vivid E9 and E95 platforms (GE Healthcare, Pewaukee, WI). Two-dimensional, color, and continuous- and pulsed-wave Doppler images were acquired from the standard acoustic windows. The severity of AS was quantified by measurement of the peak velocity across the native AV using continuous-wave Doppler to calculate peak and mean gradient, and AV area was calculated according to the continuity equation. AS patients were placed according to peak velocity, mean gradient across their native AV, baseline LVEF, and LV stroke volume index (LVSVI) into one of four previously established groups: (1) normal-flow and high-gradient (NFHG; normal LVEF, LVSVI >35 ml/m^2^, and a mean transvalvular gradient ≥40 mmHg), (2) normal-flow and high-gradient with reduced LVEF (NFHG-rEF; LVEF <50%, LVSVI >35 ml/m^2^, and a mean transvalvular gradient ≥40 mmHg), (3) “classical” low-flow and low-gradient (LFLG; LVEF <50%, LVSVI <35 ml/m^2^, and mean transvalvular gradient <40 mmHg), or (4) paradoxical low-flow and low-gradient (pLFLG; LVEF ≥50%, LVSVI <35 ml/m^2^, and mean transvalvular gradient <40 mmHg) (14). Echocardiographic variables (e.g., linear LV dimension, LVEF, stroke volume, and cardiac output) were acquired and measured as outlined in American Society of Echocardiography guidelines (15). LVSVI and LV outflow track (LVOT) cardiac index were calculated as stroke volume and cardiac output normalized to body surface area, respectively. Echocardiographic characteristics of TAVR, particularly paravalvular regurgitation, were evaluated according to previously established guidelines (16).

### GLS Analysis

LV systolic function was further evaluated by two-dimensional speckle-tracking echocardiography and assessment of peak systolic GLS. To obtain GLS, two-, three-, and four-chamber apical views were obtained on GE Vivid E9 and E95 platforms at frame rates >60 frames/sec. A region of interest was defined and manually adjusted as needed to ensure proper tracking of all segments. If one or more segments could not be adequately measured, then GLS analysis was not performed. Longitudinal systolic strain was measured in each of the 16 segments, and the software algorithm calculated an overall GLS value.

### Statistical Analysis

Continuous variables are summarized as mean ± standard deviation (SD) or median and interquartile range (IQR) as appropriate. Categorical variables are described as frequency and percentage. For two-group comparisons, unpaired t-, chi-squared, or Kruskal-Wallis tests were used. Logistic or linear regression models were used for comparisons among three or more groups. To assess LV function and outcomes, patients were divided into groups: cardiac death vs. alive and congestive heart failure hospitalization vs. no hospitalization. GLS recovery was defined as ≥15% relative increase in GLS post-TAVR, and LVEF recovery was defined as ≥10% increase in LVEF post-TAVR. Kaplan-Meier plots were used to describe time to post-TAVR survival, with the log-rank test used to compare subgroups. All tests were two-sided and *P* < 0.05 was considered statistically significant. All statistical analyses were performed in Stata (StataCorp, Version 16, College Station, TX USA).

## Results

### Baseline Characteristics

Two hundred four patients underwent TAVR during the study period. The median age of the patient population was 85 years (IQR 79–87), and 94 patients (46%) were male. The average Society of Thoracic Surgeons (STS) score was 6.9 (IQR 4.8–9.5); 80% of the patients were New York Heart Association (NYHA) functional class III/IV.

Patients were classified based on hemodynamic parameters into four subtypes of AS. There were 114 patients (56%) categorized as NFHG, 31 (15%) as NFHG-rEF, 32 (16%) as LFLG, and 27 (13%) as pLFLG. The clinical and echocardiographic characteristics of the subgroups are presented in Table 1. All subgroups were comparable in terms of comorbidities with the exception that the normal-flow AS groups (NFHG and NFHG-rEF) were more likely to have a cerebrovascular accident compared to the low-flow AS groups (LFLG and pLFLG). In terms of echocardiographic characteristics, patients in the NFHG and pLFLG AS groups showed better LV function (both LVEF and LV GLS).

**Table 1:** Patient characteristics stratified by subtypes of aortic stenosis (*n* = 204).

	**Aortic stenosis type**				
**Characteristic**	**Normal-flow, high-gradient**	**Normal-flow & high-gradient, reduced EF**	**Classical low-flow, low-gradient**	**Paradoxical low-flow, low-gradient**	
	***N* = 114**	***N* = 31**	***N* = 32**	***N* = 27**	***P-*value**
**Men**	48 (42%)	12 (39%)	25 (78%)	9 (33%)	0.001
**Age, years**	85 (79–88)	85 (81–88)	84 (80–87)	84 (80–87)	0.96
**Comorbid disease**					
CAD	78 (68%)	24 (77%)	22 (69%)	17 (63%)	0.68
COPD	44 (39%)	12 (39%)	10 (31%)	8 (30%)	0.75
CVA	22 (19%)	6 (19%)	1 (3.1%)	0	0.006
HTN	86 (75%)	22 (71%)	22 (69%)	16 (59%)	0.40
PAD	56 (49%)	12 (39%)	12 (38%)	12 (44%)	0.57
DM	39 (34%)	11 (36%)	9 (28%)	8 (30%)	0.89
**Creatinine**	1.16 (0.90–1.62)	1.23 (1.01–1.79)	1.27 (1.05–1.49)	0.95 (0.82–1.29)	0.020
**NYHA class III/IV**	90 (80%)	28 (90%)	27 (84%)	19 (70%)	0.26
**Pre-TAVR**					
LVEF	63.9 ± 7.5	38.3 ± 10.3	34.9 ± 11.0	59.1 ± 8.5	<0.0001
AV mean gradient	46.16 ± 12.67	45.49 ± 9.45	30.01 ± 9.67	32.85 ± 5.79	<0.0001
GLS	−15.65 ± 3.57	−10.84 ± 3.11	−9.50 ± 2.54	−15.35 ± 4.11	<0.0001
Stroke volume index	43.95 ± 10.26	37.60 ± 13.47	33.86 ± 8.38	33.36 ± 5.94	<0.0001
LV e' septal velocity	4.45 ± 1.45 (*n* = 94)	3.63 ± 1.34 (*n* = 28)	4.44 ± 1.35 (*n* = 15)	4.99 ± 1.98 (*n* = 21)	0.016
AVA	0.79 ± 0.33 (*n* = 107)	0.65 ± 0.27 (*n* = 30)	0.78 ± 0.21 (*n* = 29)	0.77 ± 0.17 (*n* = 25)	0.14
LA volume index	50.12 ± 16.06 (*n* = 103)	48.66 ± 15.22 (*n* = 27)	49.38 ± 8.98 (*n* = 15)	47.58 ± 21.58 (*n* = 21)	0.92
LVOT cardiac index	48.51 ± 14.23 (*n* = 103)	42.82 ± 16.68 (*n* = 30)	36.20 ± 18.16 (*n* = 26)	41.65 ± 9.85 (*n* = 25)	0.0010
PA systolic	46.95 ± 17.34 (*n* = 84)	49.75 ± 15.24 (*n* = 23)	45.10 ± 16.08 (*n* = 16)	52.71 ± 17.94 (*n* = 19)	0.48
**Post-TAVR**					
LVEF	64.7 ± 7.8	47.5 ± 12.1	38.1 ± 12.9	60.9 ± 9.3	<0.0001
AV mean gradient	10.20 ± 6.26	8.80 ± 4.49	7.47 ± 3.89	7.23 ± 3.01	0.013
GLS	−16.27 ± 3.63	−12.88 ± 4.11	−10.38 ± 2.95	−16.20 ± 3.73	<0.0001
Stroke volume index	44.60 ± 11.81 (*n* = 101)	38.64 ± 13.53 (*n* = 26)	39.88 ± 9.93 (*n* = 26)	35.77 ± 10.8806 (*n* = 24)	0.0027
LV e' septal velocity	4.73 ± 1.27 (*n* = 74)	4.78 ± 1.71 (*n* = 21)	4.40 ± 1.97 (*n* = 16)	5.83 ± 1.88 (*n* = 16)	0.041
AVA	2.00 ± 0.48 (*n* = 105)	1.96 ± 0.77 (*n* = 26)	2.11 ± 0.44 (*n* = 28)	2.01 ± 0.58 (*n* = 23)	0.75
LA volume index	49.09 ± 15.47 (*n* = 107)	48.56 ± 14.21 (*n* = 29)	53.92 ± 15.25 (*n* = 31)	49.08 ± 21.09 (*n* = 24)	0.38
LVOT cardiac index	55.21 ± 18.76 (*n* = 105)	45.09 ± 17.52 (*n* = 26)	43.85 ± 18.32 (*n* = 28)	45.06 ± 14.31 (*n* = 22)	0.0021
PA systolic	46.92 ± 15.86 (*n* = 100)	49.07 ± 11.04 (*n* = 25)	45.16 ± 14.84 (*n* = 29)	44.88 ± 15.42 (*n* = 27)	0.72
**TAVR access type**					0.83
Transfemoral	98 (86%)	26 (84%)	29 (91%)	23 (85%)	
Transapical	7 (6%)	1 (3%)	0	1 (4%)	
Direct aortic	9 (8%)	4 (13%)	3 (9%)	3 (11%)	

*Data presented as n (%), mean ± SD, or median (IQR). Where the number of events was <2, only n is given. AV, aortic valve; AVA, aortic valve area; CAD, coronary artery disease; COPD, chronic obstructive pulmonary disease; CVA, cerebrovascular accident; DM, diabetes mellitus; EF, ejection fraction; GLS, global longitudinal strain; HTN, hypertension; IQR, interquartile range; LA, center atrial; LVEF, center ventricular ejection fraction; LVOT, center ventricular outflow tract; NYHA, New York Heart Association; PA, pulmonary artery; PAD, peripheral artery disease; SD, standard deviation; TAVR, transcatheter aortic valve replacement*.

### TAVR Procedure

A transfemoral approach was used in the majority of patients (176 patients, 86%). CoreValve and Evolut R valves (Medtronic, Minneapolis, MN) were deployed in 185 patients (91%), and the Sapien XT valve (Edwards Lifesciences Corp., Irvine, CA) was utilized in 19 patients (9%). All patients underwent a successful TAVR procedure, and access site and type of TAVR valve used were comparable among AS subgroups. At post-TAVR follow-up, there was reduction of AV mean gradient (pre 41.8 ± 12.9 mmHg, post 9.12 ± 5.5 mmHg, Δ −32.7 ± 13.2 mmHg, *P* < 0.0001) and increase in AV area (pre 0.77 ± 0.29 cm^2^, post 2.01 ± 0.54 cm^2^, Δ 1.24 ± 0.64 cm^2^, *P* < 0.0001). Improvement in AV mean gradient was expectedly higher in the normal-flow AS groups than the low-flow AS groups (Δ −36 vs. −24 mmHg, *P* < 0.0001). The valve area was comparable among subgroups.

### Change in LV Systolic Function Post-TAVR

Among the entire cohort, LVEF (Δ 2.6 ± 9.3%, *P* = 0.0001) and GLS (Δ −0.91 ± 3.10, *P* < 0.0001) improved significantly. The greatest improvement in LVEF and GLS was seen in patients with NFHG-rEF AS (Δ GLS −2.05 ± 3.42, *P* = 0.02, Δ LVEF 9.2 ± 10.6, *P* < 0.0001). A significant improvement in LVEF was demonstrated in both the normal-flow AS groups (Δ 2.6 ± 9.3%, *P* = 0.001) and low-flow AS groups (Δ 2.6 ± 9.4%, *P* = 0.040). A similar pattern of improvement was seen in GLS, with significant improvement in the normal-flow AS groups (Δ −0.93 ± 3.10, *P* < 0.001) and improvement in the low-flow AS groups (Δ −0.87 ± 3.10, *P* = 0.036).

GLS recovery was not associated with coronary artery disease (66 vs. 71%, *P* = 0.5) or hypertension (68 vs. 74%, *P* = 0.3). GLS recovery was associated with effective orifice area indexed to body surface area (EOAi). Those with GLS recovery had higher EOAi post-TAVR (1.2 ± 0.37 cm^2^/m^2^) than those without GLS recovery (1.0 ± 0.29 cm^2^/m^2^, *P* = 0.0025). GLS recovery was associated with lower patient-prosthesis mismatch (defined as EOAi <0.85 cm^2^/m^2^). Only 15% of patients with GLS recovery had patient-prosthesis mismatch vs. 32% of patients without GLS recovery (*P* = 0.016).

### Clinical Outcomes

There were no major procedural complications (including cerebrovascular accident, major vascular complications, and operative death) at the time of intervention. One patient in the NFHG AS group and one in the NFHG-rEF AS group required a pacemaker at discharge. The median length of hospital stay was six days (IQR 4–8) and was similar across all subgroups. At one year, 32 of the 204 patients (16%) had died, with 11 (5%) of these deaths due to cardiac causes. Subgroup analysis at one year showed cardiac mortality was higher in the low-flow AS groups than in the normal-flow AS groups (14 vs. 2%, *P* = 0.003). At one year, hospitalization for congestive heart failure occurred in 30% (*n* = 61) of the overall study population. It was lowest in the NFHG AS group (24%) and was more than 30% in the rest of the subgroups (Table 2).

**Table 2:** Clinical outcomes stratified by subtypes of aortic stenosis (*n* = 204).

	**Aortic stenosis type**				
**Characteristic**	**Normal-flow, high-gradient**	**Normal-flow & high-gradient, reduced EF**	**Classical low-flow, low-gradient**	**Paradoxical low-flow, low-gradient**	
	***n* = 114**	***n* = 31**	***n* = 32**	***n* = 27**	***P*-value**
Length of stay (days)	6 (5–8)	7 (5–9)	6 (4–8.5)	6 (4–7)	0.65
Death in 1 year	14 (12%)	4 (13%)	7 (22%)	7 (26%)	0.23
Cardiac death in 1 year	2 (2%)	1 (3%)	6 (19%)	2 (7%)	0.004
CHF hospitalization in 1 year	27 (24%)	11 (36%)	14 (44%)	97 (33%)	0.13

*Data presented as n (%) or median (IQR). CHF, congestive heart failure; IQR, interquartile range*.

**Figure 1:**
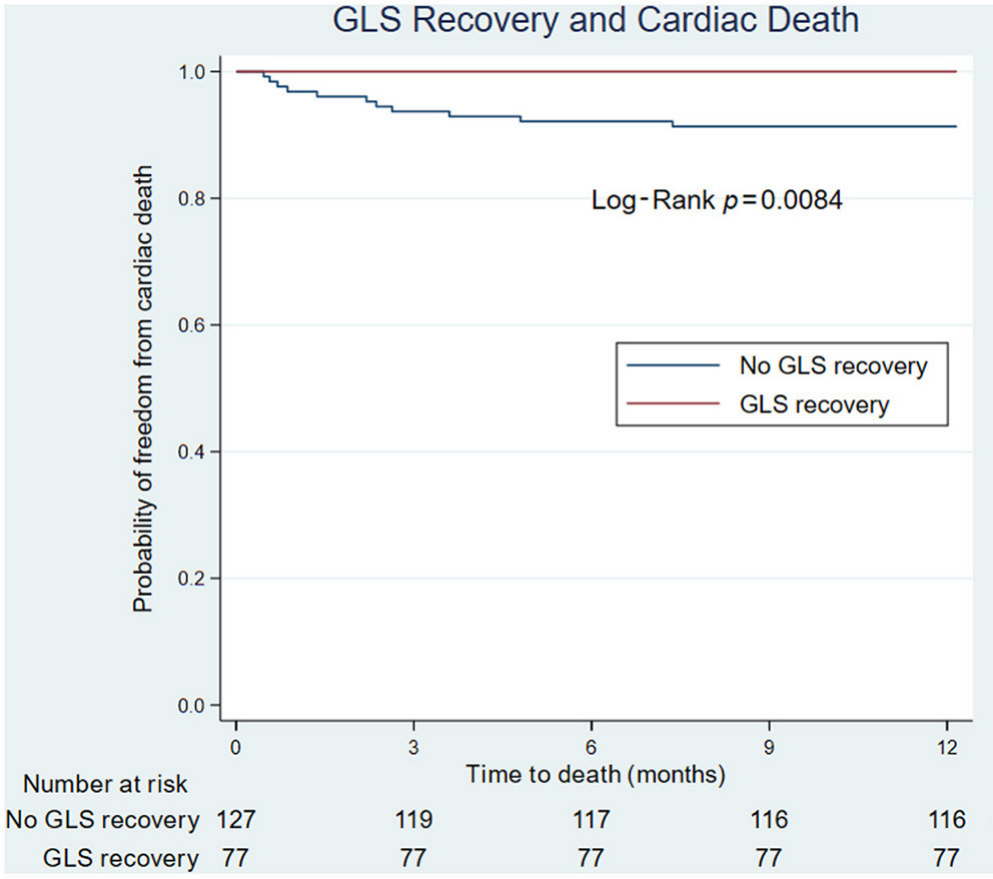
Forest plot of echocardiographic variables that predict cardiac death or congestive heart failure hospitalizations. CHF, congestive heart failure; GLS, global longitudinal strain; LVEF, left ventricular ejection fraction; OR, odds ratio; SD, standard deviation; TAVR, transcatheter aortic valve replacement.

To assess how LV function affected outcomes, patients were divided into two groups based on one-year mortality due to cardiac causes (Table 3). GLS recovery did not occur in patients who died due to cardiac causes during the first year, whereas the opposite was true in the survivors (Δ 2.0 ± 2.1 vs. −1.1 ± 3.1, *P* = 0.002). A similar pattern was seen in LVEF (Δ −4.5 ± 9.7 vs. 3.0 ± 9.2, *P* = 0.009). All patients without GLS recovery and LVEF recovery died within one year due to a cardiac cause. Post-TAVR GLS, the overall change in GLS, post-TAVR LVEF, and the overall change in LVEF were significantly associated with cardiac mortality (Figure 1). Patients were then divided into two groups based on one-year congestive heart failure hospitalization. There was no significant difference in either GLS recovery or LVEF recovery between groups (Table 3).

**Table 3:** Baseline characteristics for TAVR patients who were alive vs. those who died of cardiac causes at 1 year and those hospitalized for CHF vs. those with no CHF hospitalization at 1 year.

	**Cardiac death**	**Alive**	**OR (95% CI)**	***P*-value**	**CHF hospitalization**	**No hospitalization**	**OR (95% CI)**	***P*-value**
*N*	11	193			61	143		
Age (per 10 years)	85 (79–87)	84 (79–88)	1.32 (0.50, 3.47)	0.58	82 (79–87)	85 (80–88)	0.94 (0.63, 1.40)	0.75
Male sex	9 (82%)	85 (44%)	5.72 (1.20, 27.2)	0.028	29 (48%)	65 (45%)	1.09 (0.60, 1.98)	0.78
Pre-GLS	−13.4 ± 4.1	−13.9 ± 4.3	0.97 (0.84, 1.12)	0.69	−13.6 ± 4.7	−14.0 ± 4.1	0.97 (0.91, 1.05)	0.48
Post-GLS	−11.4 ± 3.7	−15.0 ± 4.2	0.81 (0.69, 0.95)	0.010	−14.2 ± 4.5	−15.1 ± 4.1	0.95 (0.89, 1.02)	0.19
Change in GLS	2.0 ± 2.1	−1.1 ± 3.1	0.71 (0.57, 0.89)	0.002	−0.63 ± 3.4	−1.0 ± 3.0	0.96 (0.87, 1.06)	0.40
Pre-LVEF	48.5 ± 14.2	55.2 ± 15.1	0.97 (0.94, 1.01)	0.16	51.6 ± 16.1	56.2 ± 14.5	0.98 (0.96, 0.999)	0.047
Post-LVEF	43.9 ± 14.6	58.2 ± 13.7	0.94 (0.90, 0.98)	0.003	53.4 ± 15.3	59.1 ± 13.2	0.97 (0.95, 0.99)	0.008
Change in LVEF	−4.5 ± 9.7	3.0 ± 9.2	0.91 (0.84, 0.98)	0.009	1.8 ± 9.9	2.9 ± 9.1	0.99 (0.95, 1.02)	0.42

*Data presented as n (%), mean ± SD, or median (IQR). CHF, congestive heart failure; CI, confidence interval; GLS, global longitudinal strain; IQR, interquartile range; LVEF, left ventricular ejection fraction; OR, odds ratio; SD, standard deviation; TAVR, transcatheter aortic valve replacement*.

**Figure 2:**
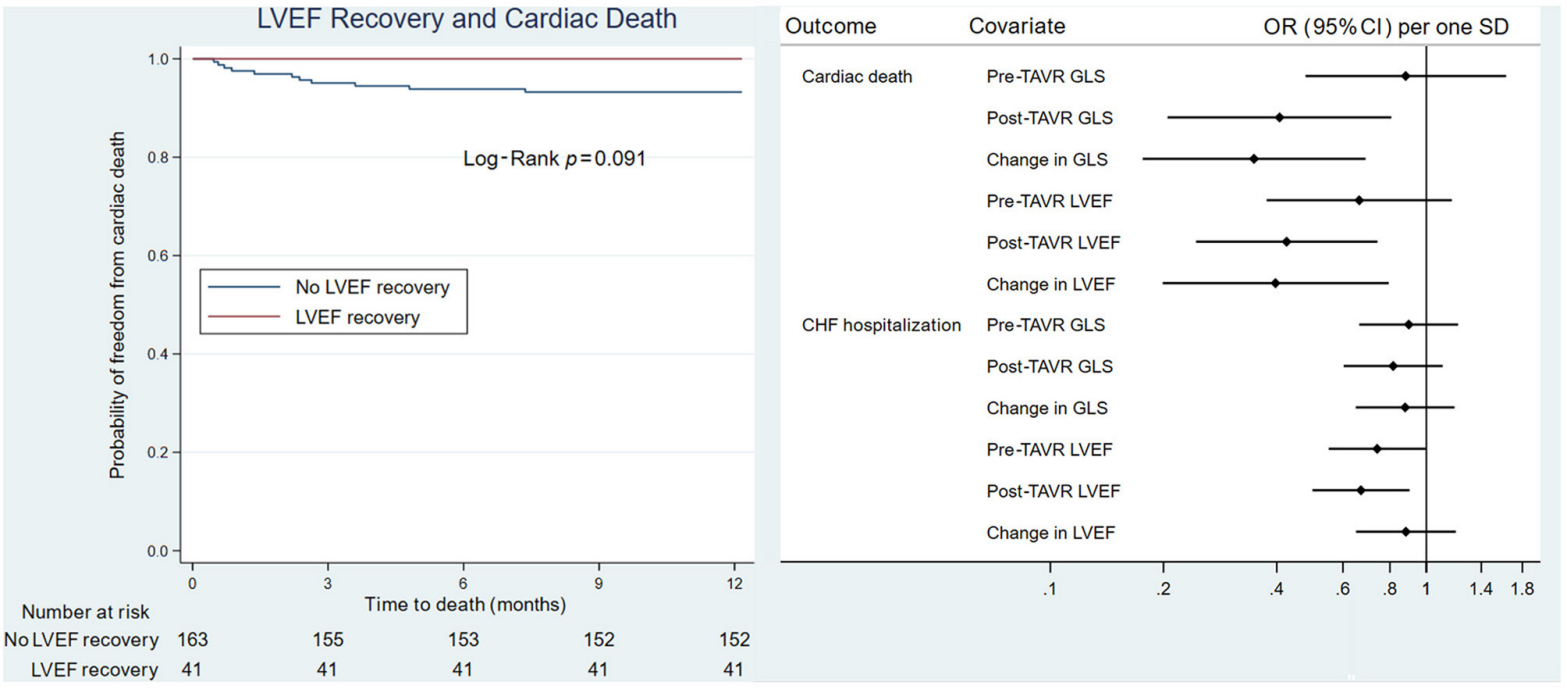
Kaplan-Meier survival analysis of GLS recovery and LVEF recovery. **(Left)** GLS recovery associated with cardiac death. **(Right)** LVEF recovery not associated with cardiac death. GLS, global longitudinal strain; LVEF, left ventricular ejection fraction.

On univariate analysis of cardiac death, male sex (HR 5.5, CI 1.19, 25.42, *P* = 0.029) and post-TAVR GLS (HR 1.22, CI 1.05, 1.42, *P* = 0.009) were significantly associated with poor cardiac survival. For every 1 percentage point worsening in GLS, the odds of death increased by 1.4 (IQR 1.1–1.8). On Kaplan-Meier survival analysis, improvement in LVEF did not predict cardiac death; however, lack of GLS recovery was associated with cardiac death (Figure 2).

## Discussion

Our study demonstrates the following findings: (1) GLS is abnormal in severe AS across all subtypes, (2) GLS does not return to normal values one month post-TAVR, suggesting that adverse LV remodeling persists even after relief of the stenotic valve, (3) improvement of GLS occurs in normal-flow AS groups and low-flow AS groups, with the greatest improvement seen in the NFHG-rEF AS group, and (4) improvement in GLS post-TAVR is directly related to survival from cardiac mortality—for every 1 percentage point worsening in GLS, the odds of death increased by 1.4 (IQR 1.1–1.8).

GLS is a well-known prognostic marker in severe AS. GLS detects subclinical dysfunction and has incremental prognostic value over traditional risk markers, including hemodynamic severity, symptom class, and LVEF in patients with AS. Prior studies have shown that abnormal GLS is a subclinical marker of LV dysfunction in severe AS even when there is no change in LVEF (17, 18). Kempny et al. (8) importantly observed a longitudinal strain of −13.3% as the optimal cutoff value, with a sensitivity of 66.7% and a specificity of 86.3%, for predicting post-interventional GLS normalization. In their study, change in GLS correlated with improvement in NYHA class but did not show significant association with major adverse cardiovascular events or 30-day mortality. D'Andrea et al. (19) calculated an LV GLS cutoff of −12.0% to identify LFLG AS patients with lack of remodeling post-TAVR. A recent meta-analysis observed a cutoff of −14.7% as a trigger to consider early interventions (20). In our study of severe AS patients presenting with TAVR, mean GLS was uniformly abnormal (−13.9% ± 4.3), although more abnormal in low-flow AS groups.

LV dysfunction detected by GLS in patients with AS has been correlated with fibrosis on endomyocardial biopsies, indicating myocardial damage, presumably from chronic increased afterload. After AV replacement, longitudinal strain improved in those with less fibrosis; in those with more extensive fibrosis, longitudinal strain did not improve (21). Puls et al. (22) performed myocardial biopsies at the time of TAVR in 100 patients. They noted that patients with myocardial fibrosis had increased cardiovascular mortality post-TAVR compared with those patients with no myocardial fibrosis (26 vs. 2%, *P* = 0.00003). Thus, myocardial fibrosis, correlated to GLS, is associated with cardiovascular death. We noted similar findings in our study: cardiovascular death was associated with lack of GLS recovery post-TAVR. Myocardial fibrosis represents an end-state pathway in myocardial damage.

These findings likely explain the heterogeneity in GLS recovery post-TAVR. This has been studied in prior literature. At an intermediate follow-up of three months post-TAVR, Giannini et al. (12) and Al-Rashid et al. (23) demonstrated an improvement in longitudinal strain despite any significant change in LVEF. Lozano et al. (24) and Alenezi et al. (25) demonstrated an improvement in GLS at one year post-TAVR with no significant change in LVEF, suggesting strain may be a more sensitive measure for changes in LV recovery.

Kamperidis et al. (26) studied 68 patients with LFLG AS post-TAVR. They reported significant improvement in GLS over a period of one year; the improvement was the greatest in the first six months post-TAVR and then plateaued. The improvement in GLS was influenced by baseline GLS and patient prosthesis mismatch, a finding different from our study, in which baseline GLS did not predict GLS recovery or clinical outcomes. We noted similar findings with regard to patient-prosthesis mismatch in our study, with greater GLS recovery in patients without patient-prosthesis mismatch post-TAVR. Løgstrup et al. (27) assessed 100 patients undergoing TAVR and found that GLS improved in patients with baseline LVEF >50% and improved but was still impaired in patients with LVEF <50%. In our study, we demonstrated immediate improvement of GLS post-TAVR, with the greatest improvement seen in the normal-flow AS groups compared with the low-flow AS groups. This differential improvement of GLS suggests that a low-flow state indicates advanced AS with underlying myopathy due to negative remodeling. This cardiomyopathy does not immediately resolve even with resolution of increased afterload post-TAVR.

There are several limitations to this study, including the retrospective nature of the review and it being a single-center study. The sample size is also a limitation, although the total sample size is large, a few of the subgroups are relatively small. The patients were selected based on availability of pre- and post-TAVR GLS measurements. The time interval from TAVR to follow-up (one month) may be too short to evaluate the complete recovery of LVEF, GLS recovery, and possible reverse remodeling.

In a recent study by Vollema et al. (28), LV GLS was associated with all-cause mortality independent of the stage of cardiac damage, and, when included in the classification, it showed incremental prognostic value over clinical characteristics and staging of cardiac damage. In conclusion, GLS is an important adverse prognostic marker in severe AS. Yet, fixing AS by TAVR does not normalize GLS. In smaller studies, baseline GLS has improved post-TAVR but still remained impaired. In our study, we noted this finding in low-flow AS groups. Lack of GLS recovery was associated with cardiovascular death. From this, we can surmise that GLS is a marker of severe damage in AS that is often irreversible. Future research is needed to determine if there are other, earlier imaging markers of subclinical LV dysfunction that can be reversed with treatment of AS (29).

## Data Availability Statement

The original contributions presented in the study are included in the article/supplementary material, further inquiries can be directed to the corresponding author/s.

## Ethics Statement

The studies involving human participants were reviewed and approved by Aurora Health Care Institutional Review Board. Written informed consent for participation was not required for this study in accordance with the national legislation and the institutional requirements.

## Author Contributions

TB, KA, SA, SD, BK, AT, and RJ conceived of and designed the study. DH and SD acquired the data. AW, DH, SK, KA, SA, SD, BK, AT, and RJ analyzed and interpreted the data. AW and SK wrote the initial manuscript. DH, TB, SK, KA, SA, SD, BK, AT, and RJ revised and reviewed the manuscript. All authors have given final approval to the version submitted and agree to be responsible for the content of the work.

## Conflict of Interest

The authors declare that the research was conducted in the absence of any commercial or financial relationships that could be construed as a potential conflict of interest.

## Publisher's Note

All claims expressed in this article are solely those of the authors and do not necessarily represent those of their affiliated organizations, or those of the publisher, the editors and the reviewers. Any product that may be evaluated in this article, or claim that may be made by its manufacturer, is not guaranteed or endorsed by the publisher.
